# Active Quantum Biomaterials‐Enhanced Microrobots for Food Safety

**DOI:** 10.1002/smll.202404248

**Published:** 2024-10-24

**Authors:** Jyoti, Alberto‐Rodríguez Castillo, Beatriz Jurado‐Sánchez, Martin Pumera, Alberto Escarpa

**Affiliations:** ^1^ Department of Analytical Chemistry, Physical Chemistry, and Chemical Engineering Universidad de Alcala Alcala de Henares Madrid E‐28802 Spain; ^2^ Future Energy and Innovation Laboratory Central European Institute of Technology Brno University of Technology (CEITEC‐BUT) Brno 61200 Czech Republic; ^3^ Chemical Research Institute “Andres M. Del Río,” Universidad de Alcala Alcala de Henares Madrid E‐28802 Spain; ^4^ Advanced Nanorobots & Multiscale Robotics Laboratory Faculty of Electrical Engineering and Computer Science VSB‐Technical University of Ostrava 17. Listopadu 2172/15 Ostrava 70800 Czech Republic; ^5^ Department of Medical Research China Medical University Hospital China Medical University No. 91 Hsueh‐Shih Road Taichung 40402 Taiwan

**Keywords:** affinity peptide, endotoxins, fluorescence, microrobots, quantum materials

## Abstract

Timely disruptive tools for the detection of pathogens in foods are needed to face global health and economic challenges. Herein, the utilization of quantum biomaterials‐enhanced microrobots (QBEMRs) as autonomous mobile sensors designed for the precise detection of endotoxins originating from *Salmonella enterica (S. enterica)* as an indicator species for food‐borne contamination globally is presented. A fluorescent molecule‐labeled affinity peptide functions as a specific probe, is quenched upon binding to the surface of QBEMRs. Owing to its selective affinity for endotoxin, in the presence of *S. enterica* the fluorescence is restored and easy to observe and quantifies optical color change to indicate the presence of *Salmonella*. The devised approach is designed to achieve highly sensitive detection of the *S. enterica serovar Typhimurium* endotoxin with exquisite selectivity through the utilization of QBEMRs. Notably, no fluorescence signal is observed in the presence of endotoxins bearing similar structural characteristics, highlighting the selectivity of the approach during food sample analysis. Technically, the strategy is implemented in microplate readers to extend microrobots‐based approaches to the routine laboratory. This new platform can provide fast and anticipated results in food safety.

## Graphical abstract

The article discusses the use of quantum biomaterials‐enhanced microrobots for rapid detection of *Salmonella enterica* endotoxins in food. These microrobots function as mobile sensors using fluorescence‐based technology for high selectivity and sensitivity. This method offers faster, more efficient, and cost‐effective detection compared to traditional techniques, making it valuable for improving food safety and preventing contamination.

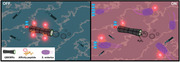

## 1 Introduction

Food safety is a paramount concern in today's world, with an increasing global population and globalization of the food supply chains. The proliferation of food‐borne pathogens is a growing matter for public health and the food industry. *Salmonella* is an anaerobic, gram‐negative, rod‐shaped bacterium that readily spreads among humans and animals, leading to food and waterborne diseases.^[^ ^1^ ^]^ *Salmonella* *typhimurium* (*S. typhimurium*) is the principal representative among a cluster of bacterial species responsible for numerous cases of food‐borne illnesses (diarrhea, abdominal pain, nausea) each year, leading to hospitalizations and millions of dollars in annual economic losses worldwide.^[^ ^2^, ^3^, ^4^ ^]^ In 2022, the World Health Organization (WHO) reported 34 instances of food safety incidents related to *Salmonella* across member states and territories.^[^ ^5^ ^]^ Among *Salmonella* infections, the serovar *S. typhimurium* had the highest incidence rate, linked to nosocomial infections and severe food poisoning with a substantial fatality rate.^[^ ^6^ ^]^ This data underscores the significance of addressing *Salmonella*‐related risks to public health. To combat such risks, the European Union has implemented strict standards to ensure *Salmonella*‐free food.^[^ ^7^ ^]^ These regulations define the permissible values that vary depending on the type of food.^[^ ^8^ ^]^ However, the data underscore the importance of addressing *Salmonella*‐related risks to public health and emphasizes the need for innovative and rapid methods of identifying and mitigating *Salmonella* contamination in food products. The traditional culture‐based methods are time‐consuming, thus preventing fast action.^[^ ^9^ ^]^ Alternatives, such as polymerase chain reaction (PCR)^[^ ^10^, ^11^ ^]^ and enzyme‐linked immunosorbent assays (ELISA),^[^ ^12^, ^13^ ^]^ offer high reliability and low limits of detection, but are laborious and time‐consuming, and require specialized equipment and personnel. Therefore, it is crucial to develop prompt, robust, and cost‐effective novel methods for real‐time detection of *Salmonella* contamination and other food‐related pathogens.

Over the past decade, there has been significant progress in biosensing technologies and the development of new biomaterials.^[^ ^14^, ^15^, ^16^, ^17^ ^]^ These advancements have been effectively applied for the detection of biomarkers associated with major food‐borne pathogens in a myriad of biodefense, food safety, and public health settings.^[^ ^18^, ^19^, ^20^, ^21^ ^]^

Quantum materials (QMs) have paved the way for groundbreaking developments in food safety. In this context, particularly the emerging field of “active quantum biomaterials” is revolutionizing the way we approach food safety. Quantum dots are 0D (carbon) nanostructures that exhibit quantum confinement effects, leading to size‐dependent electronic properties^[^ ^22^ ^]^ that make them ideal carriers for optical biosensors. Furthermore, these QMs can significantly enhance the capabilities of biosensors due to their unique physicochemical properties and diverse surface chemistry.^[^ ^23^, ^24^, ^25^, ^26^, ^27^ ^]^Chemically propelled microrobots, powered by chemical energy sources, are micromachines capable of maneuvering through complex environments. This enables them to precisely reach predetermined locations for targeted sensing. Particularly noteworthy is their proficiency in *on‐the‐fly* bio‐recognition, enabling rapid interaction with target substances and eliminating the need for additional steps in the detection process, resulting in a significant reduction in overall costs. Owing to these exceptional benefits, microrobots are employed in various fields such as catalysis, environmental remediation, cancer therapy, and biosensing among other applications,^[^ ^28^, ^29^, ^30^, ^31^, ^32^ ^]^ with enormous potential in food safety diagnosis, an unexploited area by microrobots.

Given the potential of QMs for biosensing, our research group explored the integration of such materials as active elements in polycaprolactone/Pt catalytic Janus microrobots for fluorescence detection of *Escherichia coli* (*E. coli*) endotoxins in clinical samples, including human serum and urine^[^ ^33^ ^]^ as well as *S. enterica* endotoxins in milk, eggs, and mayonnaise.^[^ ^34^ ^]^ A notable drawback of this method lies in its constrained selectivity and specificity. This approach predominantly depends on recognizing the oligosaccharide core, a shared fragment present in endotoxins across most gram‐negative bacteria. Later, strategies were implemented by our research group for the use of selective affinity peptides in *OFF‐ON* approaches but using chalcogenides as active materials for encapsulation into the micromotors.^[^ ^35^, ^36^ ^]^ QMs can be also used as the external layer of catalytic tubular microrobots prepared by template‐assisted electrodeposition. The resulting QBEMRs have a high density of active functional groups for probe immobilization, combining enhanced fluid mixing and motion in microvolume samples.^[^ ^37^ ^]^ The “moving biosensors” with optimal analytical features, have been already illustrated by our research group for the detection of DNA. The outer QMs layer allows for the incorporation of fluorescein amidite‐labeled, DNA probe by π–π interactions. In the presence of the target DNA, the probe is released, increasing the fluorescence of the solution for *OFF‐ON* detection and allowing detection at the nM range.^[^ ^38^ ^]^ In all these strategies, only a few microliters of sample are required, achieving detection in a few minutes.

This study aims to demonstrate the huge potential of QBEMRs for food safety applications related to the detection of *S. enterica serovar typhimurium* endotoxin as a highly relevant bacterial toxin in the field of food safety while limiting the required sample volumes.

## 2 Results and Discussion

**Scheme 1:**
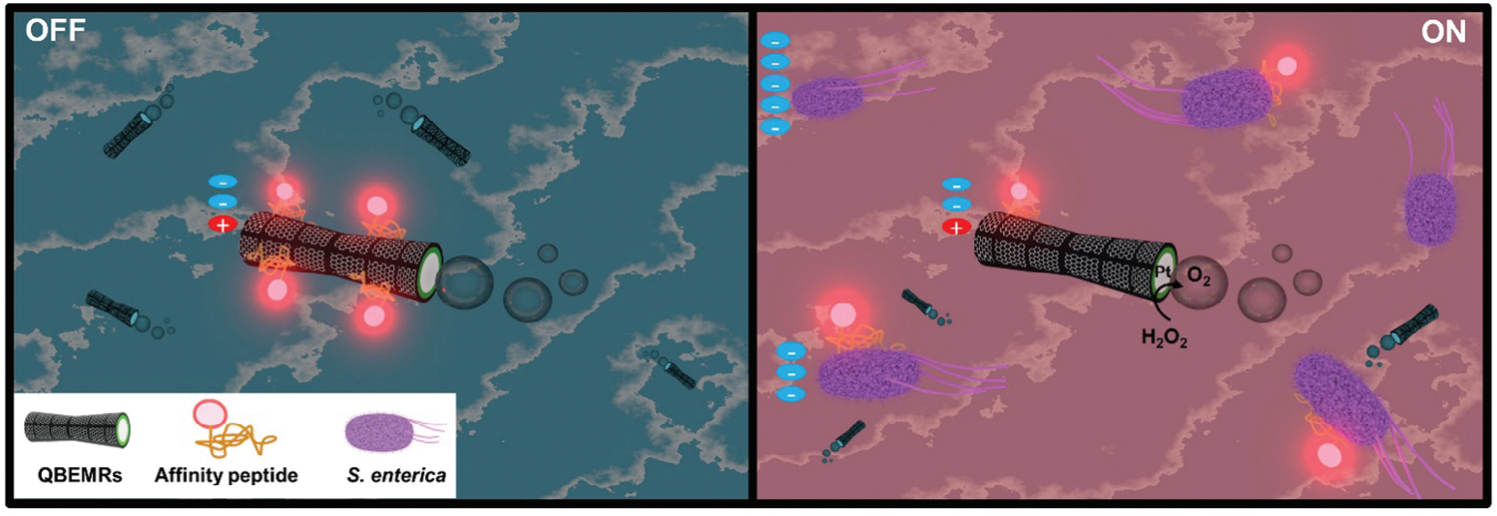
Self‐propelled graphene‐based QBEMRs as active biocarriers for *on‐the‐fly* detection of *S. enterica* endotoxin. The scheme shows the QBEMRs with the attached Rhodamine‐labeled affinity peptide before (*OFF* in solution) and after (*ON* in solution) navigation in an *S. enterica* endotoxin‐contaminated solution. This initiated the release and interaction of the affinity peptide with the *S. enterica* endotoxin, resulting in fluorescence recovery in the solution (*ON* stage). (For relative charge symbols, see Figure 2A).

An *OFF–ON* fluorescence detection strategy is employed for the sensitive detection of low levels of endotoxins by functionalization of the surface of the microrobots with a fluorescence‐labeled affinity peptide as described in **Scheme** **1**. The presence of sp^2^ groups in the outer QBEMRs’ surface facilitates the immobilization by π stacking. In the presence of the target endotoxin, the peptides are released from the microrobots’ surface, increasing the fluorescence of the solution in a concentration‐dependent manner. The strategy is implemented in a microplate reader for high‐throughput analysis. By harnessing the properties of quantum biomaterials and the agility of micro robots, these innovative systems promise precision, speed, and versatility.

### 2.1 QBEMR Synthesis, Functionalization, and Characterization

**Figure 1:**
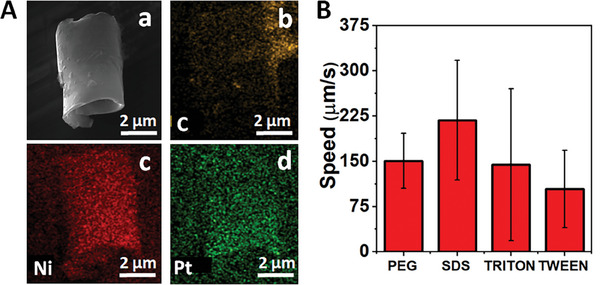
A)‐(a) SEM and EDX (b–d) images of the QBEMRs. (B) Effect of the surfactants on the speed of the QBEMRs at 9% H_2_O_2_ levels and 1% of each surfactant. Error bars represent the standard deviation of three measurements.

We prepared microrobots by electrodeposition technique. To confirm the successful synthesis of microrobots and elucidate their surface morphology and composition, SEM‐EDX was conducted. **Figure** **1**A‐a showcases the SEM images of the microrobots, revealing a tubular structure with an approximate length of 5 µm and a cross‐sectional diameter of 2.5 µm. The elemental mapping in Figure 1A, such as (b), (c), and (d), indicates the presence of key elements, notably C, Ni, and Pt. These findings suggest the successful synthesis of self‐propelled microrobots and the presence of three distinct layers: QMs, Ni, and Pt as the outer, middle, and inner layers, respectively.

The outer QMs‐based layer serves as the chemically active surface. Due to its exceptional chemical reactivity and unique electronic properties, it is a suitable choice for facilitating various chemical reactions and interactions at the material's surface.^[^ ^39^, ^40^ ^]^ Hence, after the addition of the fluorescence‐labeled affinity peptide, quenching of the solution fluorescence was observed due to the electrostatic interactions between the GQMs and the peptide,^[^ ^21^, ^41^ ^]^ Nickel (Ni), on the other hand, forms the magnetic core, constituting the middle layer of this composite structure. The presence of Ni in this configuration likely imparts magnetic properties to the material, potentially enabling it to respond to magnetic fields and exhibit magnetic behavior, facilitating the washing steps. These properties hold considerable promise when exploring alternative propulsion schemes, desirable in labile samples.^[^ ^42^, ^43^ ^]^ Platinum (Pt) serves as the innermost layer in this composite material, functioning as a motion inducer. It is strategically placed within the inner wall of the tubular configuration to initiate motion. This placement allows for a precise and controlled mechanism for inducing motion. Within this tube, Pt undergoes nucleation, maturation, and diffusion, ultimately expelling oxygen from one of the micromotor's openings and propelling it in the opposite direction, which triggers dynamic motion in the microtube as required for specific applications.^[^ ^44^, ^45^ ^]^

Before employing these microrobots for analyte detection, we investigated the impact of various surfactants on the speed as depicted in Figure 1B. It was observed that in the absence of surfactants, microrobots were unable to achieve self‐propulsion, especially at lower concentrations of H_2_O_2_. This highlights the essential role of surfactants in lowering the surface tension of the microrobots and stabilizing the generation of bubbles, which ultimately improves the micromotor speed.^[^ ^46^, ^47^, ^48^ ^]^ Hence, to optimize the concentration of H_2_O_2_, we explored various surfactants, specifically anionic (sodium dodecyl sulfate, SDS) and nonionic (polyethylene glycol, PEG, Tween 20, Triton X‐100) surfactants at 1% concentration. Notably, we found that the use of 1% SDS resulted in significantly higher microrobot speed. In contrast, the use of nonionic surfactants, such as PEG, Tween 20, and Triton X‐100, resulted in a significant decrease in micromotor speed, even with high variability in the case of Triton X‐100. This reduction in speed can be attributed to the nonionic surfactants’ larger molecular mass, which is ≈4 times that of the other surfactants used, and it substantially slowed the microrobots.^[^ ^47^, ^48^, ^49^ ^]^ Yet, in further studies, 1% PEG solution was used to block potential nonspecific binding of fluorescent biomolecules to the microrobots, minimizing the background noise and, thus, enhancing performance in the analytical detection step.^[^ ^50^ ^]^

Once the synthesis and motion of QBEMRs were confirmed, we studied the presence of surface functional groups and successful functionalization by FTIR spectroscopy. The FTIR spectra of the microrobots revealed the characteristic absorption bands corresponding to the stretching and bending vibration of the aromatic C─H group at 3385 cm^−1^, C = C stretching at 1637 cm^−1^, C─H aromatic at 2000 cm^−1^, and epoxy stretching vibration of C─O─C groups at 1049 cm^−1^ (as shown in Figure S1, Supporting Information), whereas the peaks (i.e., located ≈654, and 3400 cm^−1^, corresponding to N─H amines) were attributed to the presence of amino groups from peptide.^[^ ^51^, ^52^, ^53^ ^]^ Additionally, the presence of C = O stretching at 1650 cm^−1^ and C─N group at 1250–1020 cm^−1^ confirmed the conjugation (peptide@QBEMRs). Moreover, the slight shift of the peptide@ QBEMRs’ spectrum toward the lower wavenumbers also confirmed conjugation.

**Figure 2:**
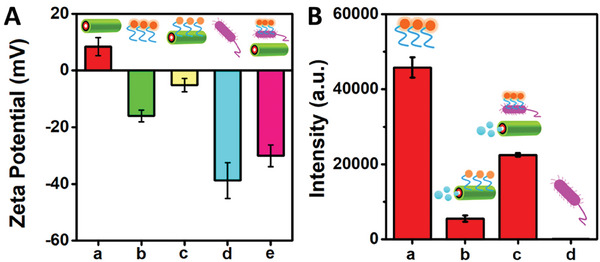
A) Z‐potential measurements of GQDs and peptide@QBEMRs biocarriers before and after *S. enterica* endotoxin interactions: a) QBEMRs; b) RhO‐labeled affinity peptide; c) peptide@QBEMRs; d) *S. typhimurium* endotoxins; e) peptide@QBEMRs interacting with *S. typhimurium* endotoxins. B) Histogram illustrating fluorescence intensity controls for (a) RhO‐labeled affinity peptide, b) peptide@QBEMRs, c) peptide@ QBEMRs interacting with *S. typhimurium* endotoxins, d) *S. typhimurium* endotoxins. Experimental conditions: λ_ex_ = 560 nm; [peptide, *S. enterica* endotoxin] = 50 µg mL^−1^; incubation time: 10 min; recovery time: 5 min; 1% PEG; 9% v/v in H_2_O_2_. Error bars represent the standard deviation (*n *= 3).

Next, we investigated the charge distribution of QBEMRs at two key stages: *before* and *after* biofunctionalization. This was accomplished through Z‐potential measurements conducted under various conditions, which included examination of the bare QBEMRs and the peptide@QBEMRs along with affinity peptide and endotoxin as shown in **Figure** **2**A. It was observed that the zeta potential of the peptide@QBEMRs decreased to −5.12 ± 2.37 mV following the successful functionalization of the positively charged QBEMRs (+8.38 ± 3.18 mV) and the affinity peptide, which carried a negative charge of −16.04 ± 2.02 mV. This shift in Z‐potential aligns with the anticipated electrostatic interactions between the microrobots’ surface and the affinity peptide. Subsequently, upon the introduction of the negatively charged endotoxin (−38.79 ± 6.33 mV), the overall charge of the sample containing these dynamic biocarriers increased to −30.07 ± 3.81 mV. This confirmed the detachment of the affinity peptide from the QBEMRs’ surface, thus demonstrating the effective *OFF‐ON* interactions of the affinity peptide with the microrobots’ surface. It is noteworthy that these tests were conducted with microrobots in a stationary state (*no fuel*, e.g., H_2_O_2_).

Consequently, to characterize the observed *OFF‐ON* behavior of the affinity peptide after the introduction of *S. typhimurium* endotoxin, an optical measurement was conducted. Figure 2B illustrates the fluorescence measurement controls. Figure 2B‐a) depicts the fluorescence intensity of the affinity peptide only. Following the incubation of the affinity peptide with the QBEMRs there was a significant decrease in fluorescence intensity (Figure 2B‐b). This decrease in fluorescence can be related to the interaction of the affinity peptide with the microrobots’ surface, thus causing fluorescence quenching (*OFF state*), whereas the introduction of the definite endotoxin, due to the high binding affinity of the affinity peptide to the *S. typhimurium* endotoxins, caused the peptide to dissociate from the QBEMRs’ surface. As follows, it leads to the restoration of fluorescence of the fluorophore as depicted in Figure 2B‐c). Almost no fluorescence was observed from the separate *S. typhimurium* endotoxins control (Figure 2B‐d). Thus, motivated by these promising results, we further exploited these active quantum biocarriers for the detection of such endotoxins in food samples.

### 2.2 Optical *OFF‐ON* Determination of Endotoxin Using QBEMRs

Before moving to the analyte concentration calibration study, it is important to highlight that several essential optimization studies have been conducted, including the peptide loading efficiency of the QBEMRs and the incubation time optimization study for peptide@QBEMRs complexes as illustrated in Figure S2 (Supporting Information). Optimization is considered as Equation (1):

(1) $$\eta \%=\left(\left[Fo-Fx\right]/Fo\right)*100$$

which calculates the loading efficiency expressed as a percentage of a substance (η%), typically a peptide, onto QBEMRs. *Fo* is the initial fluorescence of the affinity peptide, serving as a reference. *Fx* is the fluorescence of the peptide following incubation with QBEMRs, reflecting the effectiveness of the substance loading onto the carrier. A higher η% indicates more efficient loading of affinity peptide onto the active biocarriers as shown in Table S1 (Supporting Information). Based on the study results, we concluded that incubating QBEMRs with a 50 µg mL^−1^ peptide concentration for 10 min was the optimum time to carry out the subsequent studies. The 50 µg mL^−1^concentration was selected for further studies, as it provides the best compromise between peptide‐loaded efficiency and system performance. Although 10 µg mL^−1^ had the highest efficiency, it was expected that it may provide insufficient peptide coverage. Higher concentrations (75 and 100 µg mL^−1^) displayed a decrease in efficiency, meaning saturation and insufficient utilization of peptides. Hence, 50 µg mL^−1^ with 54% loading efficiency was selected so that enough peptide is loaded without wastage and optimum performance of the sensor is assured. The incubation with the concentration 50 µg mL^−1^ led to the best loading efficiencies; however, once released, the lower fluorescence intensity due to the relatively low amount of peptide used resulted in a decrease in sensitivity and low reproducibility. Furthermore, we performed a comprehensive investigation into the analyte detection time (0, 2, 5, 7, 10, 15, and 18 min), i.e., for *S. typhimurium* endotoxin (as presented in Figure S3, Supporting Information) and determined 5 min to be the optimal duration as longer times did not increase the signal significantly.

**Figure 3:**
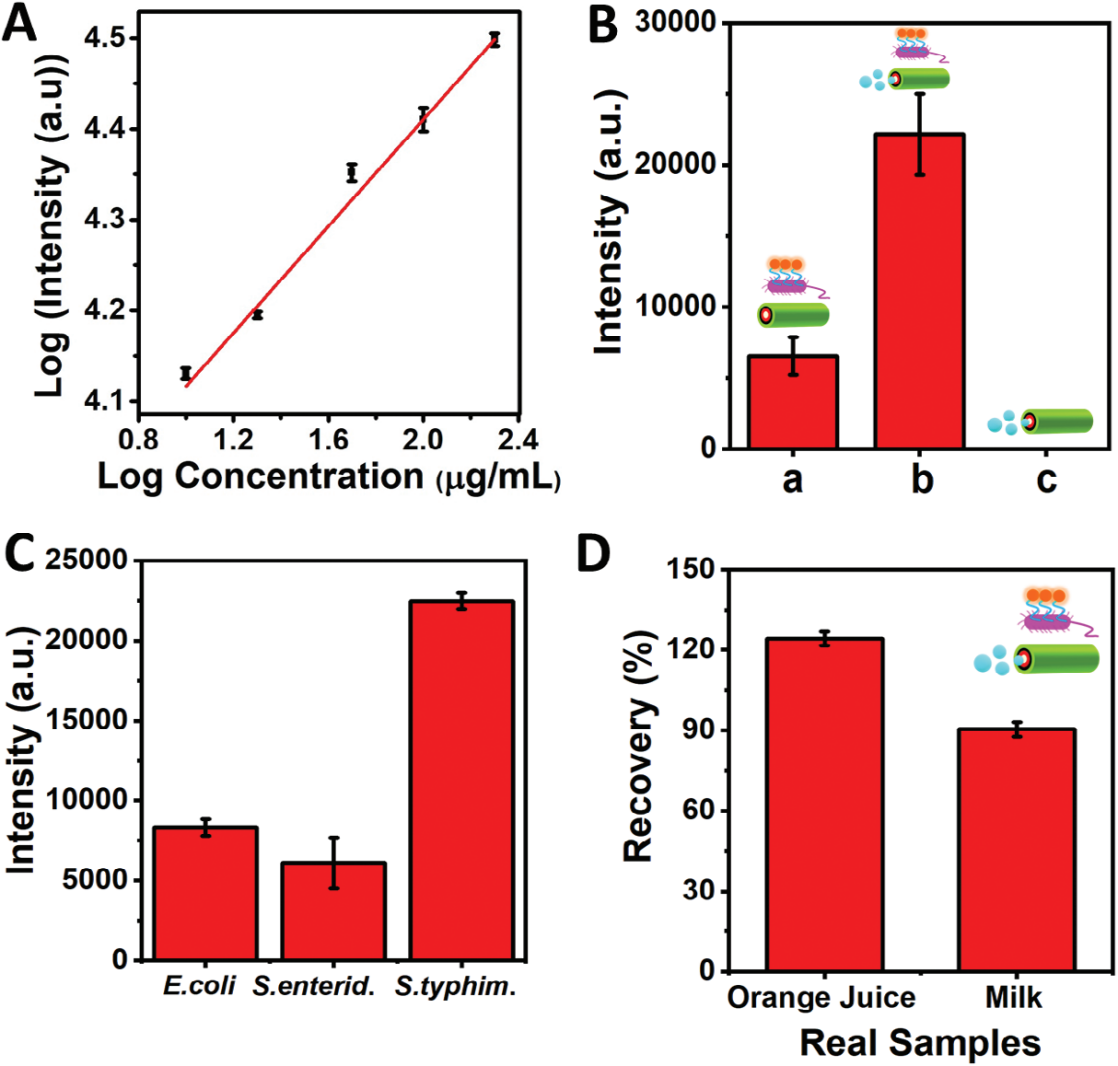
QBEMRs active biocarriers toward the optical *on‐the‐fly S. enterica* endotoxin determination. A) Calibration plot between the logarithmic (log) values of the concentration of the analyte [endotoxin] versus log [Fluorescence Intensity] under motion, with its corresponding error bars (*n = 3*). B) Histogram illustrating the recovery in the fluorescence intensities achieved by the peptide@QBEMRs toward a fixed concentration of *S. enterica* endotoxins (50 µg mL^−1^) under motion (9% H_2_O_2_) or static (no fuel) conditions. C) Histogram depicting the recovery of fluorescence intensities achieved by the peptide@QBEMRs biocarriers toward different targets (i.e.*, E. coli*, *S. enterica*, and *S. typhimurium*: (50 µg mL^−1^) (9% H_2_O_2_). D) Histogram showing the recovery obtained through the peptide@QBEMRs in various real samples (orange juice, milk) fortified with 15 µg mL^−1^ endotoxin (*n = 3*). Experimental conditions: λ_ex_ = 560 nm; incubation time: 10 min; recovery time: 5 min; 1% PEG; 9% v/v in H_2_O_2_.

Once these parameters were established, the results from these studies were implemented for the utilization of the QBEMRs for calibration performance of the *S. typhimurium* endotoxins. To achieve this, we performed a fluorescence assay within a 96‐well plate. This involved the introduction of incubated peptide@QBEMRs (90 µL) containing 1% PEG (1.5 µL). Subsequently, *S. typhimurium* endotoxin was added (45 µL) and allowed to incubate for 5 min. Followed by the introduction of H_2_O_2_ (13.5 µL) to reach a 9% H_2_O_2_ (v/v) concentration, activating real‐time detection of *S. enterica Typhimurium*. In this context, the detection approach relies on monitoring the fluorescence recovery of a quenched fluorophore (peptide@QBEMRs) as it binds to different concentrations of *S. typhimurium* endotoxin.^[^ ^3^ ^]^ Therefore, the introduction of endotoxin induces the detachment of the affinity peptide from the QBEMRs’ surface. This detachment enables the peptide to bind to the endotoxin, resulting in the recovery of fluorescence in the fluorophore in a concentration‐dependent manner. **Figure** **3**A displays a linear plot between the values of log [Fluorescence Intensity] versus the logarithmic (log) concentration of the analyte [endotoxin] with its corresponding error bars (*n = 3*). This calibration curve exhibited: *y* = 0.2893x + 3.8369, linearity with *r* = 0.990 at the 10–300 µg mL^−1^ range, boasting a remarkably low detection limit of 2.0 µg mL^−1^ and quantification limit of 6.0 µg mL^−1^. Figure 3B depicts a comparative study of the microrobots in both motion and stationary states. Notably, there was a significant reduction in the fluorescence recovery, when the microrobots were stationary (—*no fuel*) as shown in Figure 3B‐a), in contrast to their motion (—*fuel used*) in Figure 3B‐b). It can be attributed to the higher detection capabilities observed when the microrobots are actively moving. Interestingly, almost no fluorescence was detected with blank microrobots, indicating their lack of inherent fluorescence as evident in Figure 3B‐c). This was further validated by the microscopic fluorescence images as shown in Figure S4 (Supporting Information). Next, the selectivity of the active biocarriers was evaluated by exposing them to a range of gram‐negative bacteria, including *E. coli*, *S. enterica serovar Enteriditis* (*S. Enterid*), versus *S. enterica* serovar *Typhimurium* as depicted in Figure 3C. Notably, *S. enterica* Typhimurium showed stronger fluorescence recovery compared to *E. coli* and *S. enterica* *S. Enterid*. This difference in response can be attributed to variations in the structural characteristics of the endotoxin's antigen O segment, thus enabling the distinction between 50 µg mL^−1^ *S. enterica* serovar Typhimurium endotoxins versus *S. enterica* *S. Enterid*. Hence, the strategy demonstrates exquisite selectivity by employing specific affinity peptides to discriminate between bacterial types.

Subsequently, to confirm its utility in food samples such as orange juice, and milk, we conducted real‐time *on‐the‐fly* monitoring of *S. enterica* serovar Typhimurium endotoxin. For this investigation, the actual samples (orange juice, milk) were fortified with 15 µg mL^−1^ of the target endotoxin. The results, as presented in Figure 3D, showcased varying recovery percentages for each sample. Relatively good recoveries were obtained in food samples and were highly reproducible, allowing reliable *S. enterica* detection in food samples. Such recoveries reflect the complexity of the samples, which were processed untreated. This is also noticed in the significant reduction in microrobot speed, with high variability observed (≈51 ± 34, and 44 ± 25 µm s^−1^ upon the introduction of samples like orange juice, and milk). This decrease in speed was likely due to the higher viscosity of these samples as depicted in Figure S5 (Supporting Information). It is worth noting that this speed reduction did not impede the practical use of microrobots as they still achieved good recoveries in both situations. This result confirms the successful implementation of these active biocarriers for the S*. enterica* serovar Typhimurium endotoxin detection.

Furthermore, Table S2 provides a comprehensive comparison of methods developed for the detection of *S. enterica* endotoxins. First, Deby Choi et al. reported the detection of the lipopolysaccharide (LPS) antigens from *S. enterica* Typhimurium by using ELISA. While detection can be performed at the ng mL^−1^ level, long analysis times are required (4 h), along with the use of expensive and labile antibodies.^[^ ^54^ ^]^ Sannigrahi et al.^[^ ^55^ ^]^ and Wang et al.^[^ ^56^ ^]^ reported magnetic immunoassays for *S. enterica* Typhimurium endotoxin, decreasing the detection time from 30 min to 3 h, respectively. Yet, the use of impedance spectroscopy hampers the application of the method for routine analysis, representing as well high complexity. There is no question that micromotor‐based approaches improved the analysis times. Indeed, Pacheco et al.^[^ ^34^ ^]^ reported detection by using GQDs Janus micromotors as sensing probes with a lower limit of detection but an analysis time of ≈15 min. Pacheco et al. also reported the optical detection of *S. enterica* endotoxins by using transition metal dichalcogenides (TMDs) modified with affinity peptides.^[^ ^35^ ^]^ While TMDs like WS_2_ and MoS_2_ have shown promising results, TMD‐based micromotors can be sensitive to changes in environmental conditions, such as pH and temperature, thus posing a stability issue.^[^ ^57^ ^]^ Especially, food samples often contain a diverse range of compounds and some might interfere with the detection process.^[^ ^58^, ^59^, ^60^, ^61^ ^]^ TMD‐based micromotors may exhibit issues related to long‐term stability, affecting their shelf life and reliability over extended periods.^[^ ^62^, ^63^ ^]^ In addition, in the latter work using TMDs,^[^ ^35^ ^]^ the complex endotoxin‐released peptide cannot diffuse into the solution and the fluorescence increase should be measured inside the micromotor (instead of in the solution) with a high‐performance optical microscope, which prevents its implementation in microplate readers. However, QBEMRs offer high biocompatibility, ease of functionalization, and excellent stability under various conditions, contributing to the reliability of detection methods over time.^[^ ^64^, ^65^ ^]^ Hence, this approach possesses essential bioanalytical characteristics, such as high sensitivity and selectivity, rapidity, simplicity, and cost‐effectiveness, which effectively render it an efficient method for detecting *S. enterica* in food and environmental samples.

## 3 Conclusion

Our study highlights the potential of quantum biomaterials‐enhanced microrobots in connection with self‐propelled behavior to enhance sensitivity and signal stability, particularly for in‐field *S. enterica* detection in the context of food safety. Quantum biomaterials‐enhanced microrobots, when combined with affinity peptides, eliminate the need for lengthy incubation times and expensive antibodies, improve efficiency, and, thus, improve cost‐effectiveness. This method achieves a low limit of detection with a rapid detection time and low sample volumes with a high‐throughput analysis using the microplate reader. Furthermore, this method demonstrates remarkable specificity, surpassing prior techniques and displaying heightened selectivity for *S. enterica* endotoxins. We demonstrate the application of these emerging quantum materials‐enhanced microrobots in the detection of toxins in real food samples. Phage display technology can be used to search for any type of endotoxin for bacterial species. The amino acid sequence can be tailor‐made commercially and labeled with any type of fluorophore, enabling the detection of multiple species and even multiplexed assays. This quantum‐enhanced technology paves new ways for on‐demand, point‐of‐care detection of food toxins for greater food safety and security.

## 4 Experimental Section

### Chemicals

Citric acid (cat. 251275), nickel tetrahydrate (cat. 262277), nickel chloride hexahydrate (cat. N6136), boric acid (cat. 15665), chloroplatinic acid hydrate (cat. 520896), SDS (cat. 71727), PEG (cat. 89510), Tween 20 (cat. P9416), hydrogen peroxide (30%, cat. 216763) (H_2_O_2_), Triton X‐100, and endotoxins from *S. enterica* Typhimurium (cat. L6143) and *S. enterica* Enteritidis (cat. L7770) were purchased from Merck (Madrid, Spain). Rhodamine (RhO)‐labeled affinity peptide (RhO‐NFMESLPRLGMH) was custom‐made by Quimigen (Madrid, Spain).

### Equipment

Scanning electron microscopy with energy‐dispersive X‐ray detection (SEM‐EDX) characterization was performed using a TESCAN LYRA 3 XMH instrument. Further, a VERTEX 70v FT‐IR spectrometer was used to validate the interactions between the microrobots and the affinity peptide. To evaluate the charge distribution of the microrobots, Z‐potential measurements were conducted with a Malvern Zetasizer. Fluorescence measurements were performed with a Biotek Cytation 5 imaging reader at room temperature. A Nikon ECLIPSE Ti‐S/L100 inverted microscope equipped with a multi‐LED light illumination source (CoolLED's pE‐4000), a Zyla sCMOS camera, and a G‐2A epifluorescence filter (510–560 nm) were used to capture the autonomous motion behavior of the microrobots and to capture fluorescence images.

### Synthesis of Graphene Quantum Dots (GQDs)

QDs were produced via direct pyrolysis using 2 g of citric acid (CA). CA was heated to 200 °C in a 5 mL beaker on a heating mantle. Within 30 min, the liquid transformed from colorless to pale yellow to orange, indicating the generation of QDs, which were subsequently dispersed in alkaline solution. For more details on the synthesis, see the previous procedure followed.^[^ ^66^ ^]^

### Synthesis of GQMs/Ni/Pt Microrobots

Microrobots were synthesized by using the previously established membrane‐assisted electrodeposition method following Scheme S1‐i (Supporting Information).^[^ ^37^, ^67^, ^68^, ^69^ ^]^ Briefly, a 100‐nm‐thick layer of gold (Au) was sputtered onto a Whatman Cyclopore polycarbonate membrane (cat. WHA70602513, purchased from Merck) with 5 µm pore size using electron‐beam evaporation (1.4 kV, 1.8 mA). This modified membrane was assembled in a custom‐made Teflon cell, using aluminum foil as the electrical contact for the working electrode. A platinum wire was used as a counter electrode and an Ag/AgCl electrode in a 1 M KCl solution was the reference electrode. The membrane‐based electrode was then integrated into a three‐electrode cell configuration, employing electrochemical depositions that were carried out using an AUTOLAB potentiostat (Metrohm). The electrodeposition process involved the following steps: i) formation of the outer GQD layer (outermost layer for biofunctionalization): GQDs were deposited from a 0.1 mg mL^−1^ dispersion in a supporting electrolyte of 0.1 m H_2_SO_4_ containing 0.5 m Na_2_SO_4_ using cyclic voltammetry. The potential window ranged from +0.3 to –1.5 V versus Ag/AgCl, with a scan rate of 50 mV s^−1^ and a total of 40 cycles; ii) deposition of the Ni middle layer (intermediate layer to track them magnetically): The Ni middle layer was deposited from 2 m nickel sulfamate, 82 mm nickel chloride, and 485 mm boric acid (pH 4) through chronopotentiometry, applying first 10 pulses of −20 mA for 0.1 s. Then, −6 mA was applied for a duration of 350 s; iii) electrodeposition of the Pt inner layer (innermost as a catalytic layer). The Pt inner layer was electrodeposited using a 4 mm H_2_PtCl_6_ solution in 0.5 m boric acid by chronoamperometry with a bias potential set to −0.4 V versus Ag/AgCl for 2000 s. Following electrodeposition, the membrane was carefully detached from the Teflon cell and thoroughly rinsed with deionized water. Subsequently, it underwent a hand‐polishing process with an alumina slurry (0.5 µm) to remove the Au layer. The membrane was then subjected to a series of washes, including 2x dichloromethane (30 min vortex, 3 min centrifuge at 7000 rpm) followed by 2x washing in isopropanol (15 min vortex, 3 min centrifuge at 8000 rpm) and, finally, 3x washing in deionized water (15 min vortex, 10 000 rpm). The resulting microrobots were stored in 1 mL of deionized water where they remained stable for 4 weeks without any changes in their properties.

### Functionalization of the GQMs/Ni/Pt Microrobots with Affinity Peptide

Following their synthesis, the microrobots underwent modification by combining 100 µL of the as‐synthesized microrobots with 50 µg mL^−1^ peptide (100 µL) and allowing them to incubate at 25 °C for 10 min at 950 rpm to initiate the electrostatic interactions between them that result in the quenching of the fluorescence of the affinity peptide (*OFF* state) as shown in Scheme 1. The resulting active quantum biocarriers were then properly washed with deionized water by centrifuging twice for 5 min at 5000 rpm and finally resuspended in 90 µL of solution containing 1% PEG.

### Microrobot Motion Study and Endotoxin Detection Assay

Microrobot motion and speed were studied by placing 1 µL of microrobot solution on a glass slide. The solution contained 1 µL of H_2_O_2_ solution (final concentration, 9%) and 1 µL of PEG solution (final concentration, 1% v/v). Videos were recorded and speed tracked using NIS Elements Advanced Research software (Nikon).

The detection assay involved mixing 90 µL of the microrobots at a final concentration of 10 microrobots µL^−1^ with 15 µL of *S. enterica* endotoxin solution to obtain a final concentration ranging from 0 to 300 µg mL^−1^, along with 45 µL of 30% H_2_O_2_ (final concentration, 9%) in a 96‐well microplate. The mixture was allowed to sit undisturbed for 5 min to allow microrobot motion in the solution. Subsequently, fluorescence was measured at 560 nm (excitation) and 601 nm (emission) (*ON* state) (see Scheme 1). Each experiment was conducted in triplicate.

### Bacteria Culture

*Escherichia coli* strain B or *Staphylococcus aureus* bacteria were cultivated in Luria–Bertani medium and incubated at 100 rpm and 37 °C for 16 h. The culture media was then collected and used to test the performance of the procedure. All the experiments followed UAH regulations, and all biosafety measurements were taken.

## Conflict of Interest

The authors declare no conflict of interest.

## Author Contributions

J. and A.‐R.C. contributed equally to this work. J. performed conceptualization, data curation, formal analysis, investigation, and visualization, wrote the original draft, and reviewed and edited the final manuscript. A.R.C performed conceptualization, data curation, formal analysis, and investigation, and wrote the review and editing. B.J.‐S. performed conceptualization, formal analysis, funding acquisition, project administration, acquired resources, and supervision, and reviewed and edited the final manuscript. M.P. performed conceptualization, formal analysis, funding acquisition, project administration, acquired resources, and supervision, and reviewed and edited the final manuscript. A.E. performed conceptualization, formal analysis, funding acquisition, project administration, acquired resources, and supervision, and reviewed and edited the final manuscript.

## Supporting information



Supporting Information

## Data Availability

The data that support the findings of this study are available from the corresponding author upon reasonable request.
