# Supplementary material for: Active Quantum Biomaterials‐Enhanced Microrobots for Food Safety
Source: Small. 2024 Oct 24;20(52):2404248. doi: 10.1002/smll.202404248 (PMC11673522; doi:10.1002/smll.202404248)
Supplement: Supplementary file 1 — Supporting Information [file SMLL-20-2404248-s001.docx]

**Supplementary Information**

**Active Quantum Biomaterials Enhanced Microrobots for Food Safety**

Jyoti,^1,2#^Alberto-Rodríguez Castillo,^1#^ Beatriz Jurado-Sánchez,^1,3*^ Martin Pumera,^2,4,5*^ and Alberto Escarpa^1,3*^

^1^ Department of Analytical Chemistry, Physical Chemistry, and Chemical Engineering, Universidad de Alcala, Alcala de Henares, E-28802 Madrid, Spain

^2^ Future Energy and Innovation Laboratory, Central European Institute of Technology, Brno University of Technology (CEITEC-BUT), 61200 Brno, Czech Republic

^3^Chemical Research Institute “Andres M. Del Río”, Universidad de Alcala, Alcala de Henares, E-28802 Madrid, Spain

^4^Advanced Nanorobots & Multiscale Robotics Laboratory, Faculty of Electrical Engineering and Computer Science, VSB – Technical University of Ostrava, 17. Listopadu 2172/15, 70800 Ostrava, Czech Republic

^5^Department of Medical Research, China Medical University Hospital, China Medical University, No. 91 Hsueh-Shih Road, Taichung, Taiwan 40402

^*^ Corresponding authors

^#^ Both authors equally contributed


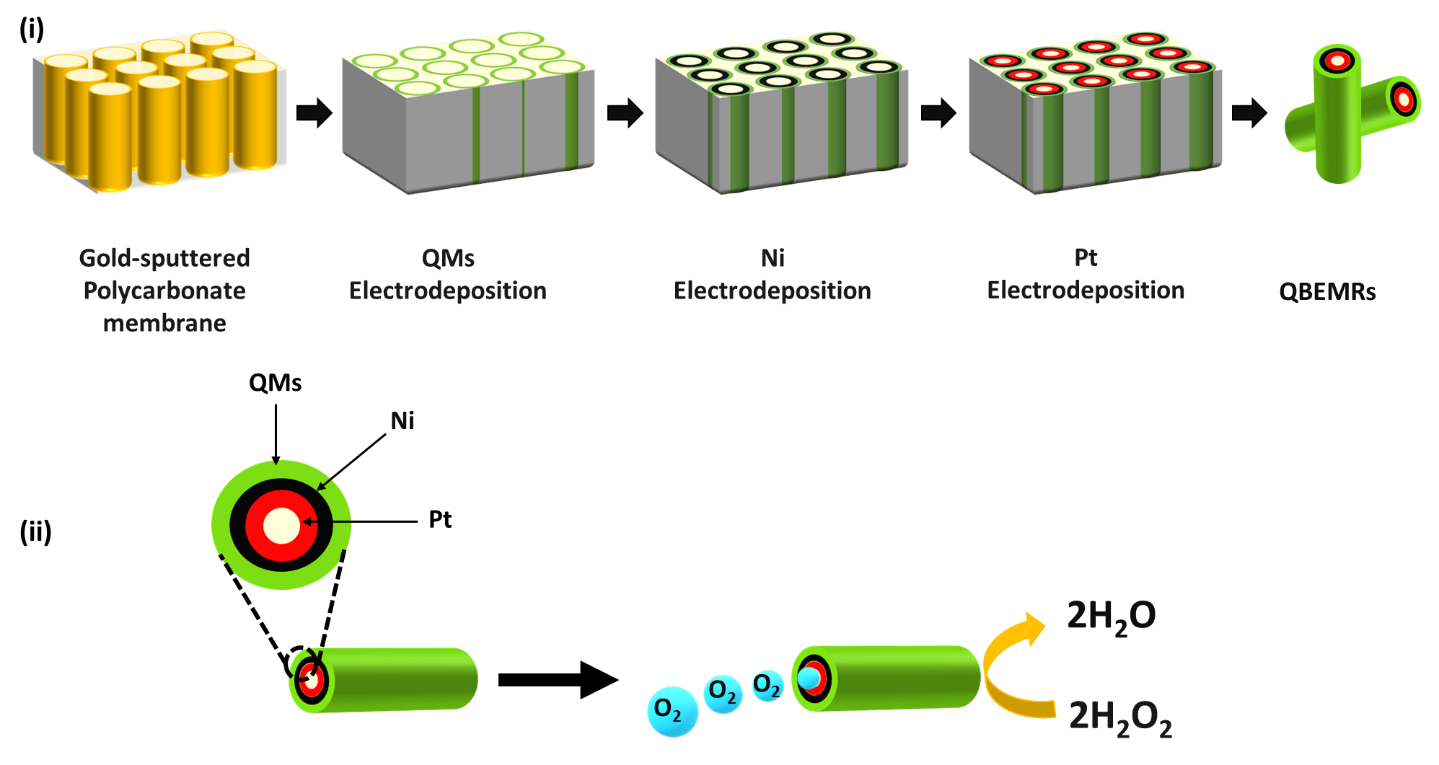


**Scheme S1. Synthesis of QMs/Ni/Pt microrobots:** Template assisted method was used to incorporate *i)* QMs (outer layer), *ii)* Ni (middle layer) and *iii)* Pt (inner layer) *via* electrodeposition.

**
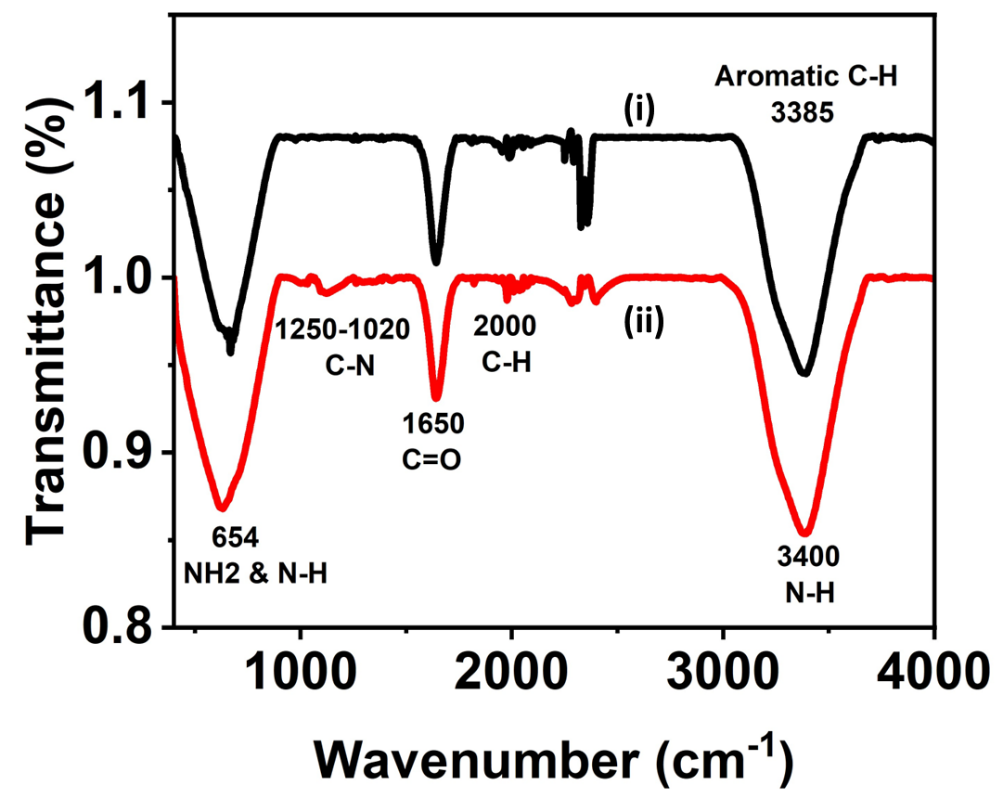
**

**Figure S1.** FTIR spectra of the **(i)** bare QBEMRs micromotors (black), **(ii)** peptide@QBEMRs micromotors (red).

**
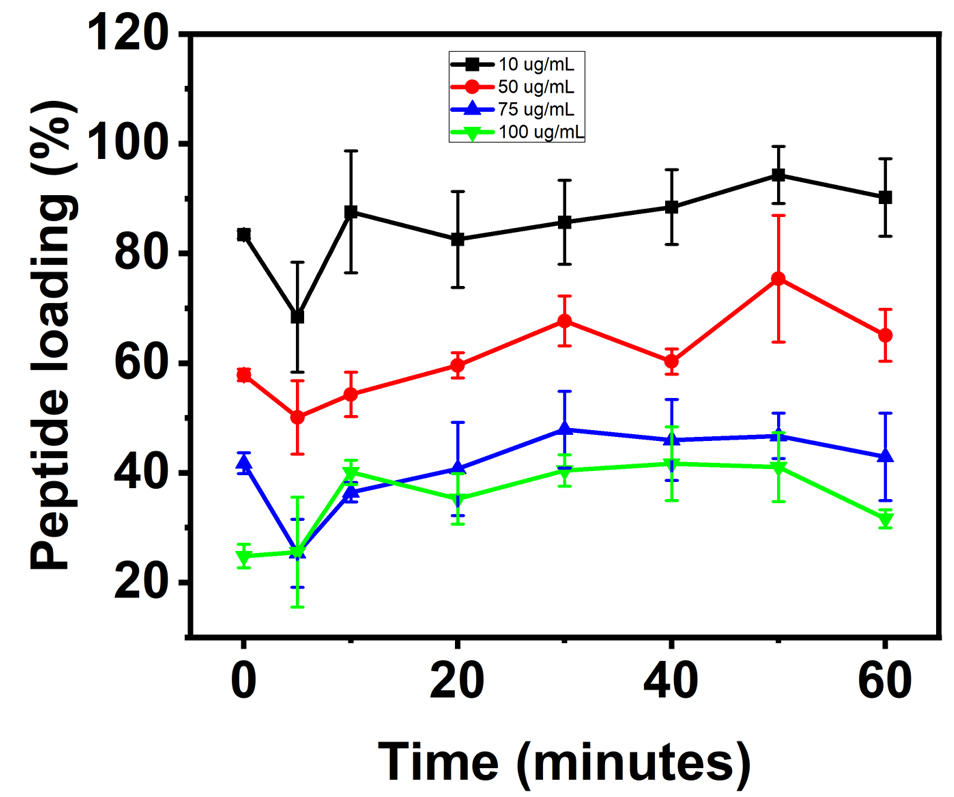
**

**Figure S2.** Optimization of the peptide loading capacity. Solutions containing different concentrations of the peptide (10-100 µg/mL) were incubated with the QBEMRs. The loading capacity was calculated by comparing the fluorescence of the solution prior and after micromotor incubation.

**Table S1.** Peptide loading efficiency at 10 minutes.

| **Concentration (µg/ml)** | **F_o_ (Affinity Peptide)** | **F_t_ (Average (n=3))** | **(F_o_-F_t_)/F_o_ *100** |
| --- | --- | --- | --- |
| 10 | 37294 | 4641 | 90 |
| 50 | 314204 | 143631 | 54 |
| 75 | 343346 | 218216 | 36 |
| 100 | 324165 | 258278 | 20 |


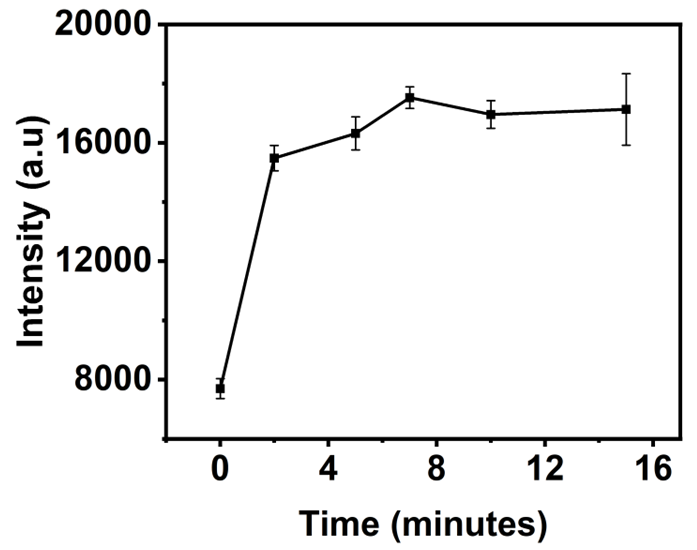


**Figure S****3.** Optimization of the detection time. Fluorescence intensity of the solution after micromotor navigation in the *S. enterica endotoxin* (50 µg/mL) solution at different times.


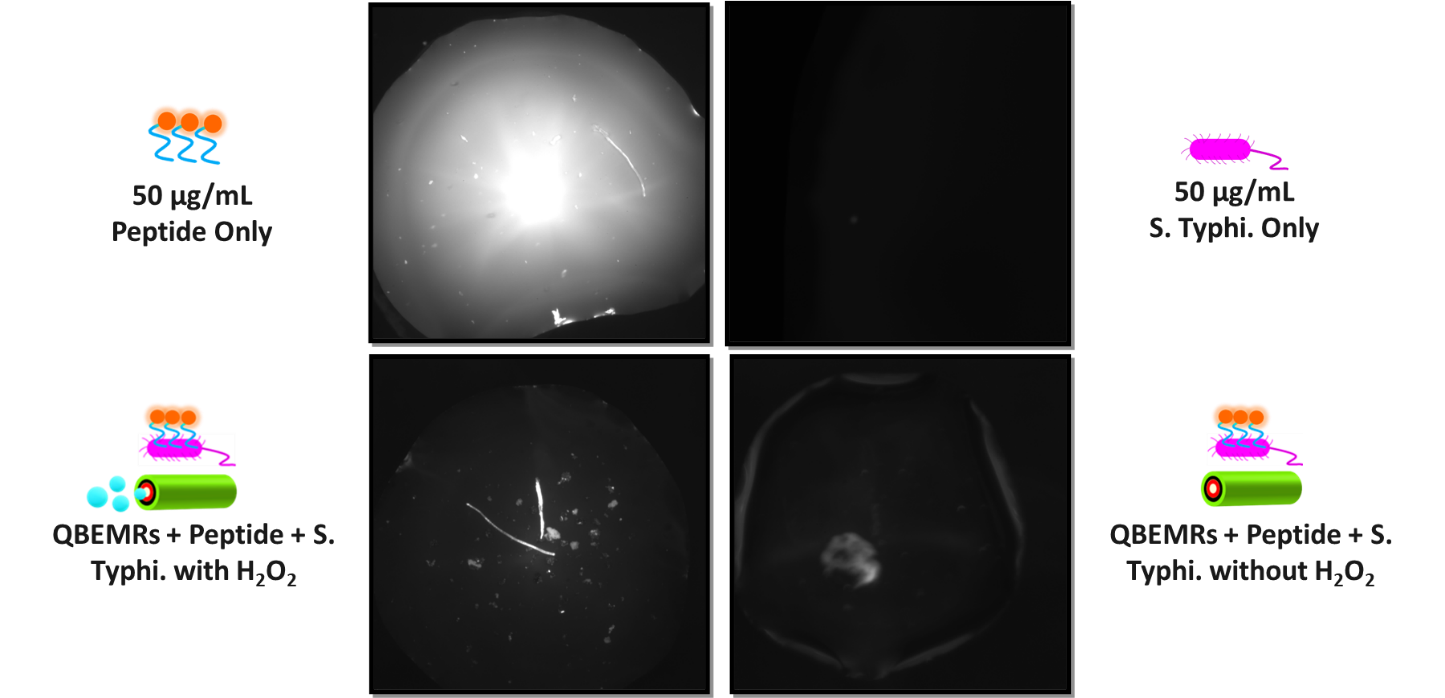


**Figure S4:** Validation by Fluorescence Images.


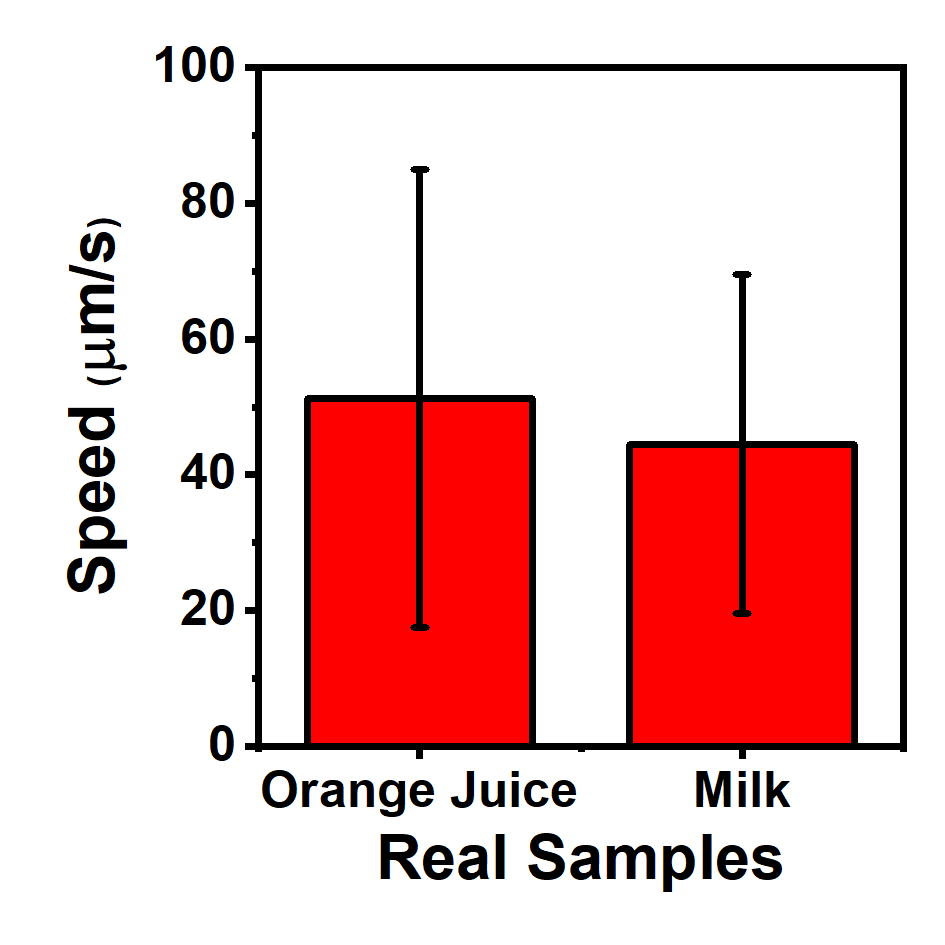


**Figure S5.** Speed of micromotors in real samples.

**Table S2.** *S. enterica* endotoxin previous literature data.

| **Approach** | **Sensing Probe** | **Detection** |  | **Analytical Performance** | | | | **Remarks** | **Ref.** |
| --- | --- | --- | --- | --- | --- | --- | --- | --- | --- |
|  |  |  | **Analyte Detected** | **Linear range** | **LOD** | **Analysis time** |  | |  |
| ELISA | Murine monoclonal antibody | Colorimetric | *S. typhimurium*;  *S. heidelberg* | NR | 1 ng mL^-1^ | 4 h | - High sensitivity - high-throughput analysis | | ^1^ |
| Magnetosome-antibody complex | Anti-Salmonella polyclonal antibody | Electrochemical impedance | *S. typhimurium* | NR | 0.001 µg mL^-1^ | 30 min | -Water and milk samples (spiked) | | ^2^ |
| Magnetic NPs-internalized macrophage cells | Murine macrophage (Ana-1) cell | Electrochemical impedance | *Escherichia coli* | 1–50 µg mL^-1^ | 0.15 µg mL^-1^ | 3 h | - High reproducibility - Real samples (spiked) | | ^3^ |
| Polycaprolactone Janus micromotors | Receptor-functionalized quantum dots | Fluorescence microscopy (ON–OFF)  Micromotors | *S. enterica* | 0.2–3.5 ng mL^-1^ | 0.07 ng mL^-1^ | 15 min | - High sensitivity - Untreated food samples (spiked) | | ^4^ |
| WS_2_ & MoS_2_ Janus micromotors | Affinity peptide | Fluorescence microscopy (OFF-ON/micromotors) | *S. typhimurium* | 4–333.3 µg mL^-1^  9.8–333.3 µg mL^-1^ | 1.2 µg mL^-1^  2.0 µg mL^-1^ | 5 min | - Low analysis time - Real, untreated samples (spiked) - Excellent recovery reproducibility | | ^5^ |
| QBEMRs | Affinity peptide | Fluorescence, microplate reader (OFF-ON/solution) | *S. typhimurium* | 10–300 μg mL^-1^ | 2.0 μg mL^-1^ | 5 min | - High especifity - High sensitivity - High-throughput analysis - Low analysis time - Untreated food samples (spiked) | | This work |

**References**

(1) Choi, D.; Tsang, R. S. W.; Ng, M. H. Sandwich Capture ELISA by a Murine Monoclonal Antibody against a Genus‐specific LPS Epitope for the Detection of Different Common Serotypes of Salmonellas. *Journal of Applied Bacteriology* **1992**. https://doi.org/10.1111/j.1365-2672.1992.tb01814.x.

(2) Sannigrahi, S.; Arumugasamy, S. K.; Mathiyarasu, J.; K, S. Magnetosome-Anti-Salmonella Antibody Complex Based Biosensor for the Detection of Salmonella Typhimurium. *Materials Science and Engineering C* **2020**. https://doi.org/10.1016/j.msec.2020.111071.

(3) Wang, X.; Zhu, P.; Pi, F.; Jiang, H.; Shao, J.; Zhang, Y.; Sun, X. A Sensitive and Simple Macrophage-Based Electrochemical Biosensor for Evaluating Lipopolysaccharide Cytotoxicity of Pathogenic Bacteria. *Biosensors and Bioelectronics* **2016**. https://doi.org/10.1016/j.bios.2016.03.007.

(4) Pacheco, M.; Jurado-Sánchez, B.; Escarpa, A. Sensitive Monitoring of Enterobacterial Contamination of Food Using Self-Propelled Janus Microsensors. *Analytical Chemistry* **2018**. https://doi.org/10.1021/acs.analchem.7b05209.

(5) Pacheco, M.; Jurado-Sánchez, B.; Escarpa, A. Transition Metal Dichalcogenide-Based Janus Micromotors for on-the-Fly Salmonella Detection. *Microchimica Acta* **2022**. https://doi.org/10.1007/s00604-022-05298-2.
